# Donation of surplus frozen pre-embryos to research in Israel: underlying motivations

**DOI:** 10.1186/s13584-016-0085-4

**Published:** 2016-11-05

**Authors:** Aviad Raz, Jonia Amer-Alshiek, Mor Goren-Margalit, Gal Jacobi, Alyssa Hochberg, Ami Amit, Foad Azem, Hadar Amir

**Affiliations:** 1 Department of Sociology and Anthropology, Ben-Gurion University of the Negev, Beer-Sheva, Israel; 2 Sarah Racine IVF Unit, Lis Maternity Hospital, Tel Aviv Sourasky Medical Center, 6 Weizmann Street, Tel Aviv, Israel; 3 Helen Schneider Hospital for Women, Rabin Medical Center, Petah Tikva, Israel

**Keywords:** Surplus frozen embryo disposition, Human embryo research, In vitro fertilization, Assisted reproductive technologies

## Abstract

**Background:**

The high number of IVF procedures performed in Israel has had an unforeseen consequence: accumulation of large amounts of surplus frozen embryos. After five years that the frozen embryos are kept for free, patients need to make an embryo disposition decision. One option is donation for research. The donation rate in Israel is very low. Our aim was to understand the attitudes, values and perceptions of female IVF patients that decided to donate their surplus frozen embryos to research.

**Methods:**

The study setting was a tertiary IVF unit which during the 2000–2009 period treated 241 patients who had their frozen pre-embryos stored for more than five years. The study population consists of the 12 patients (from among the 241) who had decided to donate their excess frozen pre-embryos to research. In-depth interviews were carried out with 8 of those 12 patients.

**Results:**

IVF patients who donated their surplus frozen pre-embryos to research viewed the frozen embryo as a valuable resource that does not have human identity yet. The majority expressed a gradualist approach to the human status of the embryo as requiring successful implantation and development in the uterus. All the respondents chose donation to research not because it was their first choice but because they did not want or were unable to use the pre-embryos in the future, in addition to not willing to thaw them. For many of the respondents, donation to research was accompanied by a sense of uncertainty. All would have preferred to donate their pre-embryos to infertile women or couples, an option which is currently prohibited in Israel.

**Conclusions:**

The moral reasoning behind decisions that patients make regarding excess pre-embryos is important for health care practitioners to consider when offering decision-making alternatives and counseling. For our respondents, the scarcity of donating excess frozen pre-embryos to research may reflect patients' preference for embryo donation to infertile couples. Recommended ways to increase donation to research may include public education and awareness, as well as targeted communication with IVF patients by multi-professional IVF unit teams comprised of a medical doctor and a professional trained in bioethics.

## Background

As in other Western countries, the expansion in the number of in vitro fertilization (IVF) procedures performed in Israel has had an unforeseen consequence: the accumulation and storage of large amounts of “surplus” frozen embryos. The number of IVF cycles in Israel totaled 34,538 in 2010 compared to nearly 32,000 during 2014 and 18,000 in 2000. Among the 34,538 cycles, embryos were transferred after test-tube fertilization in 29,961 cases [1] (http://www.jpost.com/Health-and-Science/Number-of-IVF-births-in-Israel-rises-in-last-decade). With about 10 cycles per 1000 women of reproductive age (15–49) (according to Israel's Ministry of Health), Israel has the highest number in the world of IVF treatments per capita [2], which supports the situation of a quickly growing number of surplus frozen embryos. According to Regulation 9 of the Israeli Public Health Regulations (In Vitro Fertilization) from 1987, and a Ministry of Health circular from 2008 (“Guidelines for patients on use of frozen fertilized oocytes in in vitro fertilization units”), the frozen fertilized eggs belong to both partners and may be kept for a period of five years at no cost. Later, by a written application by both partners (or of the woman only, in the case of a sperm donation), they may be kept for 5 years longer subject to payment. As a result, many IVF patients are faced with the dilemma of what to do with their frozen embryos, that is, the “disposition decision.” There are no national statistics on what happens with these leftover embryos. The couples’ decision is inherently complicated by the diversity of the potential options: the frozen embryos can be kept in storage and used by the couple in further attempts to conceive; they can be used in medical research; they can be thawed. The disposition form (documenting the couple's decision on the fate of their frozen embryos after 5 years of freezing) contains these three options in the following order: to continue the storage for fee, donation for research and discarding, with no further details. In cases where the partners disagree, the frozen embryos will remain in storage. Additionally, the case is transferred to the legal department of the hospital. If embryos are in fact used for research, after approval of the Ethics Committee, the couple will receive a detailed explanation of the study and will sign a specific consent form before the study is conducted. In other countries such as the USA, Mexico, Australia, New Zealand, Iran and Spain, an additional option is donating the excess frozen embryos to another infertile person/couple (http://www.eggdonationfriends.com; http://www.embryodonation.org.au).

The proportion of IVF patients who donate embryos to research varies greatly depending on country, from 7 % in France to 73 % in Switzerland [3] and a world-record of 92 % in Sweden, where donation to another infertile couple is not permitted and only poor-quality embryos may be donated to research [4]. Studies in the US found that for those reaching a disposition decision, donation to research was a decision made by almost 50 % of couples with frozen embryos in storage [5], while in Australia almost 30 % of such couples reported they would prefer this option [6]. It is important to note that the data presented for the various countries should be assessed carefully. These studies were carried out in a few IVF units and included up to several hundred couples/women over a relatively short time, and are therefore not nationally representative. A systematic review, published in 2014, examined factors associated with the donation and non-donation of frozen embryos for research [3]. The review included 39 studies that were derived mainly from European countries (*n* = 18) and the USA (*n* = 11). The authors found three factors associated with the donation and non-donation of frozen embryos for research: (1) prioritization of the possible options regarding embryo disposition based on patients’ beliefs about what should be done and their views regarding the moral status of embryos; (2) patients’ knowledge of benefits and risks concerning research on human embryos; and (3) patients’ experience of information exchange and trust in medico-scientific institutions. It is likely that these factors are responsible for the variety of disposition decisions in various countries. This is the first study to examine this topic in Israel.

Although the decision to donate to research can have firm motivations – commonly described by donors as better than the thawing of embryos and as an opportunity to help others or to improve health and IVF treatments [3] – some reports have shown that as many as 88 % of couples who had originally chosen to donate their embryos to research changed their minds [7]. Yet there has been scant research on how patients who have stored frozen embryos actually think about them or what are their motivations and deliberations concerning the disposition decision [8]. The impetus behind this study was to understand the motivations underlying the decision to donate to research, triggered by our initial finding that this option was the least common amongst Israeli IVF patients.

## Methods

### Design setting

Following Institutional Review Board (IRB) approval we compiled a list of IVF patients treated in Racine IVF unit, Sourasky Medical Center, between the years 2000–2009, who had their frozen pre-embryos stored for more than 5 years, and letters were sent to these women in 2008–2014.

### Participants

Table 1 describes the demographic characteristics of this group (*n* = 241) and their disposition decisions. It should be mentioned that the majority (54 %) of the larger group of IVF patients whose records were examined did not respond or their letters were returned undelivered. For the purpose of this exploratory, mixed-methods study, we used a purposive sampling frame in which a decision to donate to research was treated as a sampling category.

**Table 1:** Socio-demographic characteristics of IVF patients contacted about their surplus frozen pre-embryos

Characteristic	Total participants *n* = 241	Total patients who donated frozen pre-embryos to research *n* = 12	Total interviewees *n* = 8
Age at the time of freezing (y), mean (range)	32 (22–44)	35 (28–43)	35.6 (31–43)
Age At the time of data collection (y), mean (range)	41.5 (29–59)	44.9 (39–52)	45.8 (39–52)
Religion *n* (%)			
Jewish	196 (81.3 %)	9 (75 %)	6 (75 %)
Muslim	15 (6.2 %)	0 (0 %)	0 (0 %)
Christian	2 (0.8 %)	1 (8.3 %)	1 (12.5 %)
Unknown	28 (11.6 %)	2 (16.7 %)	1 (12.5 %)
Marital status *n* (%)			
Single	17 (7.1 %)	1 (8.3 %)	1 (12.5 %)
Married	194 (80.5 %)	8 (66.7 %)	5 (62.5 %)
Divorced	28 (11.6 %)	3 (25 %)	2 (25 %)
Widowed	2 (0.8 %)	0 (0 %)	0 (0 %)
No. of children, mean (SD)	2.3 (1.1)	2.1 (1.1)	1.9 (0.3)
Causes of infertility *n* (%)			
Male problems	101 (40.6 %)	4 (33.2 %)	3 (37.5 %)
Tubal and pelvic pathology	47 (18.9 %)	2 (16.7 %)	1 (12.5 %)
Unexplained infertility	60 (24.1 %)	2 (16.7 %)	2 (25 %)
Endometriosis	7 (2.8 %)	2 (16.7 %)	2 (25 %)
Ovulatory dysfunction	6 (2.5 %)	0 (0 %)	0 (0 %)
PGD	13 (5.2 %)	(0 %)	0 (0 %)
Fertility preservation	10 (4 %)	(0 %)	0 (0 %)
Other	5 (2 %)	2 (16.7 %)	0 (0 %)
No. of IVF cycles, mean (SD)	3 (2.5)	2.8 (1.6)	2.9 (1.9)
Fertilization *n* (%)			
Conventional IVF	57 (23.7 %)	8 (66.7 %)	5 (62.5 %)
ICSI	100 (41.5 %)	3 (25 %)	2 (25 %)
Conventional IVF + ICSI	76 (31.5 %)	1 (8.3 %)	1 (12.5 %)
Unknown	8 (3.3 %)	0 (0 %)	0 (0 %)
Origin of sperm *n* (%)			
Partner	220 (91.3 %)	8 (66.7 %)	5 (63 %)
Donor	18 (7.5 %)	3 (25 %)	2 (25 %)
Partner and Donor	0 (0 %)	1 (8.3 %)	1 (12 %)
Unknown	3 (1.2 %)	0 (0 %)	0 (0 %)
No. of frozen pre-embryos, mean (SD)	5.1 (4)	5.1 (5.1)	4.1 (5.4)
Time since the freezing (y), mean (SD)	9.3 (2.5)	10.2 (2.3)	10.2 (1.8)
Disposition decision *n* (%)			
No response	85 (35.3 %)		
Letter returned	46 (19.1 %)		
Payment for additional 5 years	38 (15.8 %)		
Defrost	26 (10.8 %)		
Frozen embryo transfer	13 (5.4 %)		
Donation to research	12 (5 %)	12 (100 %)	8 (100 %)
Request for a phone consultation	16 (6.7 %)		
Unknown	5 (2.1 %)		

### Intervention

We focused on those who decided to donate their surplus frozen pre-embryos to research – the least selected option (*n* = 12, 5 %). The prospective participants were contacted during 2015 by the IVF unit office and received a brief description of the study. Eight of the 12 agreed to participate, and they were contacted by a research assistant, signed an informed consent form, and were scheduled for an interview. Out of the remaining 4, two women refused to participate, and two women were not reachable via phone, email, or regular mail according to the details available in our unit.

Interviews were conducted, according to the participant's preference, either face-to-face (3 interviews) or through the telephone (5 interviews). Women who were interviewed face to face signed the informed consent before the interview. Women who were interviewed through the telephone sent a signed consent form via e-mail. All interviews were semi-structured; they were tape recorded and transcribed verbatim. Questions included the participants' attitudes toward their frozen pre-embryos in terms of their human status; the religious or secular aspects of their decision-making; the process underlying their decision to donate to research; their reasons for doing it; and their feelings about it. Respondents could add their own comments and concerns whenever they wanted it.

The data were analyzed by examining the transcripts for respondents’ views, descriptions, and expressions of what they considered meaningful. These responses were then broken down into discrete statements, sentences, phrases, or paragraphs that expressed an opinion, stance, feeling, or concern. We then discussed, agreed on and refined the major categories of meaning, the relationships between categories, and the development of themes, while taking into account the range and variation in the data [9]. The first thematic analysis was done by the two research assistants who conducted the interviews under the supervision of the senior co-authors, and it was then assessed for thematic saturation, confirmed and refined by the senior co-authors, with the final interpretation reflecting both the medical and sociological perspectives of the researchers. The outcomes are described in the following section.

## Results

### Demographic data

The demographic data show that the 8 respondents (presented in Table 1) were mainly Caucasian, secular, educated) level of education of 12 years or more), with no known diseases or addictions, and relatively older compared to the entire group (35.6 vs. 32 mean age in time of freezing, respectively). Two of them were originally from Russia, one from Mexico, and the others were native Israeli (except for age, all other characteristics were not available for the entire group).

By looking at the table, several factors seem to be associated with embryo donation or non-donation. Older, single women who had used sperm donation and underwent conventional IVF were more likely to donate their surplus frozen embryos for research. However, because of the small size of the sample it would be inappropriate to try and generalize from it about such factors.

In what follows we discuss three major themes that emanated from the interview analysis: the human status of the frozen embryo; motivations to donate to research; and donation of surplus frozen pre-embryos to infertile couples.

### The human status of the frozen embryo

At the beginning of the interview, although many of the respondents had no problem with the term we used ("surplus frozen pre-embryos"), some were dismayed by the term "embryo" and preferred the term "frozen fertilized eggs." Other said they prefer to use the term "pre-embryo". The very decision about which terminology to use was emotionally loaded for many of the respondents. As one of them said: "thinking about this terminology only adds to my pangs of conscience." In response to the question about when does the embryo become human, our respondents expressed a spectrum of replies – one respondent said it was as early as the moment of fertilization, while another said it was only after the first systemic ultrasound during weeks 14–16. However, for the majority of the respondents, human status required successful implantation and development in the uterus: "once it is in the uterus, it has a human status, it is completely an embryo. Emotionally as well as physiologically."

### Motivations to donate to research

Concerning their motivations to donate to research and how they think about it afterwards, our respondents expressed a shared sense of non-resolved uncertainty. All of them expressed uneasiness about their decision and many considered deciding to donate to research as a compromise, given that on the one hand, they already had all the children they wanted (or planned to have), while on the other hand, they considered the frozen pre-embryos too valuable to destroy. The option to donate to research was therefore seen as "the least bad" option. This could explain why all of the respondents said they were not interested in receiving details on the actual research their frozen pre-embryos were used in.

Even when the majority of the respondents pointed out that the decision to donate to research was propelled by wanting to contribute to the improvement of IVF treatments, this was never the whole story. The following typical quote from an interview with one of our respondents illustrates the complex personal deliberations and dilemmas behind such a decision: "yes, I donated – but then I regretted it. I really want more children, but my husband does not. Donating to science, that's very important… But I still want to have more children even though it would not happen."

### Donation of surplus frozen pre-embryos to infertile couples

All of our respondents said they would prefer to donate their surplus frozen pre-embryos to another infertile woman rather than donating them to research. As one respondent typically explained it: "donating to infertile women is probably a more important donation [compared to research]. The more I think about it, you know I am talking with you so I am thinking about it… so yes. I think that for infertile couples that have undergone treatments, it is a huge compromise to accept a baby that is not yours. Especially I know this for men. So if people have reached this impasse, in their desire to have children, it is important to help them."

All our respondents agreed that donation of surplus frozen pre-embryos to infertile couples should be made one of the disposition options in Israel. When asked what should be done with "orphan" surplus frozen pre-embryos, the majority of our respondents said that they should be donated to infertile couples. Two respondents expressed reservations concerning the complexity of such a decision, comparing it to giving a child for adoption. Indeed, all of the respondents agreed that the partner has to be consulted and that such a decision should be a couple's unanimous decision. There was a variety of views concerning the anonymity of donation. Some agreed that anonymity was important, and compared it to the situation of anonymous donor insemination. Others said, in contrast, that anonymity could be a source of incest and that donating a "whole" embryo is different than donor insemination and has to be accompanied by background information. The built-in complexity of such a process was typically illustrated by one of the respondents who said "I would be happier to know to whom I am donating… but I'm also afraid that such knowledge would be unhealthy for me, my children, and my family."

A final element complicating the disposition decision was that couples frequently conceptualized their frozen pre-embryos as symbols of the infertility that had dominated their lives for so many years. For some couples, the embryo solution was to “use them up” by having more children, while for several women the existence of the stored pre-embryos continued to fuel their desire for more children even when that longing was impractical for medical or personal reasons. For others, pre-embryos were “unfinished business” that required having to directly confront the painful memories of their own struggles with infertility. This revisiting of the infertility experience sometimes led to a consideration of embryo donation out of empathy for other infertile couples, yet couples considered embryo donation as having a meaning that was uniquely different than either gamete donation or adoption. Indeed, gamete donation, surplus embryo donation and adoption have a different meaning, in several aspects, for the donor, the recipient and the offspring. From a genetic perspective, both in embryo donation and adoption only the donors are genetically related to the embryo; while in gamete donation, in most cases half of the genetic material belongs to the recipient. Both in gamete donation and surplus embryo donation the recipient is carrying the pregnancy, but conventional wisdom is that gamete donation involves “only” oocyte/sperm while embryo donation includes a “real” baby.

## Discussion

Our findings clearly show that IVF patients who donated their surplus frozen pre-embryos to research were driven by a view of the frozen embryo as a valuable resource that does not yet have full human identity. This reaffirms and adds contextualization to previous studies showing that embryos are simultaneously perceived by patients as having a moral status as well as an instrumental value [10–12]. Other studies have also shown that life in its biological essence and in terms of being a person are not necessarily perceived as identical, although they are closely connected. The question is normally framed as whether a fertilized human egg cell/pre-embryo is a human being, and for what purpose. The Catholic Church claims human life begins at conception/fertilization, while Judaism argues for a more gradualist approach. According to Orthodox Judaism, the embryo is considered to acquire human status in a gradual manner, only after 40 days of gestation and while developing in the uterus [13]. The view of the Israeli human rights law is that a person is the subject of rights only from the moment of birth. From the perspective of lay people, it may be a biological event (e.g., implantation, fetal heartbeats, or fetal movements) that confers accumulative "personhood" on the fetus [14, 15]. Similarly to other studies that described the plurality of arguments concerning when does human life begin, our findings also demonstrate a spectrum of views, with the majority expressing a gradualist approach to the human status of the embryo as requiring successful implantation and development in the uterus – an approach which was also found as characteristic of the Israeli population in general [15, 16]. Our respondents' awareness of and emphasis on the difference between a "complete embryo" that is successfully implanted and developing and the "frozen embryo" evidently also reflected their situation as IVF patients with surplus frozen pre-embryos. This stance also played a crucial role, for our respondents, in justifying decisions such as donation to research.

Importantly, all of our respondents saw donation to research as a less than ideal option (not wanting or unable to use the pre-embryos in the future, and not willing to thaw them), and would have preferred to donate to other infertile couples. Contrary to other studies of the disposition decision that found a sense of resolution amongst their respondents [7], we found a lingering sense of discomfort and uncertainty amongst many of our respondents. This finding is in contrast to the altruistic motives cited in other studies by those who donated pre-embryos to research, and were less ambivalent about their decision than those who decided to destroy [17].

Hashiloni-Dolev and Shalom [18] found that the difficult experience of fertility treatments made many of their Israeli female respondents value the frozen embryo even more and thus being less likely to consider its donation. Our findings suggest a complementary interpretation. In fact, for all of our respondents, the difficulties experienced due to the fertility treatments led them to express a sense of identification with other infertile women and a desire to help them by donating to them the frozen pre-embryos which they did not need any more.

There are acknowledged limitations to this preliminary study. We included IVF users from 2000 onwards due to technical reasons of accuracy and completeness of the records. Only those who decided to donate their frozen pre-embryos to research were interviewed for this study. Their analysis provide insights into the participants' underlying motivations, but can provide only very limited information on why most other female IVF patients do not donate their excess pre-embryos to research. The scarcity of donating to research provided the trigger for this study, but additional types of studies are needed to really understand that scarcity. We are planning to solicit the perspectives of those who did not donate their pre-embryos to research as part of a related study.

Qualitative analysis was purposefully used in this study in order to understand the decision to donate to research from the participants' point of view. At the same time we recognize that the thematic portrayal of experience is selective. Although themes can be viewed as cultural resources that people draw on to make sense of events in their lives, in subsequent interviews people may take up different topics that reflect more recent experiences.

The motivations and views of all our respondents were secular, and many of them also expressed pro-choice views and support for organ donation. However, it should be noted that some of them also alluded to cultural themes such as the relatively liberal Jewish view of the "pre-embryo" [19]. This implies that Orthodox Jewish IVF patients could potentially decide to donate surplus frozen pre-embryos to research, and future research should address the question why they are not doing so.

## Conclusions

The impetus behind our focus on the decision to donate excess frozen pre-embryos to research was its scarcity amongst Israeli IVF patients, as compared to other Western countries. Recommended ways to increase donation to research may include, in general, public education about the importance of such research (especially in the context of infertility) and raising public awareness concerning the role of such donation in enabling specific medical research. In the specific context of the IVF unit, recommended ways to increase donation to research include providing to IVF patients more detailed and targeted information concerning donation to research through discussions with multi-professional teams comprised of a medical doctor as well as a professional trained in bioethics. It is important to note that in all the ways listed to raise awareness of the donation of frozen embryos for research, the medical staff should discuss with the couple the ethical questions involved in the donation of frozen pre-embryos for research. In addition, the health professionals and researchers should communicate realistic expectations and disseminate accurate information concerning the results from research on human embryos, so that couples will be more able to make their own (informed and reflected) decision. The couple should realize that every choice of theirs is acceptable.

Another factor that might influence donation to research, as gleaned from the interviews, is the quality of information provided about such research. According to existing procedures, the disposition decision is made without specific information about the research in which the donated embryos will be used. If embryos are in fact used for research, the couple will receive a detailed explanation of the study and will sign a specific consent form before the study is conducted. A more detailed explanation about future studies at the time of the decision could influence the couple's choice.

Our findings also draw attention to IVF patients preference for embryo donation (to infertile couples rather than research), which is prohibited in Israel. The prohibition on pre-embryo donation to infertile women exists as a general legal prohibition for Israeli donors (not for overseas fertilized egg donations), and is not explained in any Israeli official publication on the handling of frozen pre-embryos and therefore we could not provide an explanation as to why it is prohibited for *Israeli* women to donate frozen pre-embryos to other Israeli infertile women. Our findings signal an unmet desire among IVF patients that should be urgently addressed. The IVF patients with surplus frozen pre-embryos we interviewed did not want to discard them or to use them in the future, and, without an alternative, were reluctantly driven into donating them to research. Undocumented cases of implantation of surplus frozen pre-embryos at a time that is unlikely to produce a pregnancy so as to give these pre-embryos a "decent end" also demonstrate this problem. If the patient cannot be reached or does not provide a response, Ministry of Health (Form 08/2008) regulations prescribe that surplus frozen pre-embryos stored more than 10 years are defrosted by default [20]. However, according to the guidelines, this process will not begin before receiving final instructions from the Ministry of Health. In practice, no clear instructions were given until today and the IVF units continue to store these frozen embryos. This fact, too, highlights the urgent need to reconsider the importance of adding the option of embryo donation to infertile women, which could provide a solution to the needs of many IVF patients who face the disposition decision, as well as to the predicament of those in need of such a donation. While some may argue that opening up the option of embryo donation to infertile couples may reduce donation to research, in countries where these two options were suggested, the percentage of embryo donation to research has not been reduced [21–23].

## Abbreviations

ICSI, Intracytoplasmic sperm injection; IVF, In vitro fertilization; PGD, Preimplantation genetic diagnosis
